# Generation of high amplitude compressions and rarefactions in a photoacoustically excited droplet

**DOI:** 10.1016/j.pacs.2021.100289

**Published:** 2021-07-21

**Authors:** Xingchi Yan, Gerald J. Diebold

**Affiliations:** Department of Chemistry, Brown University, Providence, RI, 02912, USA

**Keywords:** Photoacoustic, Optoacoustic, Laser, Ultrasound

## Abstract

Photoacoustic excitation of a fluid sphere generates an outgoing ultrasonic wave whose time profile permits determination of the density, sound speed, and diameter of the sphere. Experiments with pulsed laser beams have confirmed the major predictions of existing theory. With regard to acoustic waves generated within spheres, although mathematical expressions for their properties are known, virtually no exploration of the waveforms in theory or experiment has taken place. Here, two cases for photoacoustic excitation of a droplet are discussed: first, absorption of radiation in a region of fluid external to the droplet, and, second, absorption of radiation by the droplet itself. Large amplitude transients, compressions in the former and rarefactions in the latter, are generated as the waves approach the center of the sphere. The high amplitudes of the waves suggest shock wave formation.

## 1 Introduction

A salient feature of the photoacoustic effect [1], [2] in fluids is that the temporal and spatial characteristics of the ultrasonic waves generated by absorption of short pulse optical radiation contains information on the spatial absorption profile of the irradiated body. This principle was first demonstrated experimentally in a series of papers where optically thin fluid and solid bodies with simple geometries immersed in various solvents were irradiated by nanosecond laser pulses. Subsequently this feature of the photoacoustic effect has been used as the basis for medical imaging [3], [4], [5], [6], [7], [8], [9], [10], [11], [12] where arrays of transducers are used to record the spatial and temporal patterns of emitted acoustic waves with sophisticated mathematical methods employed to invert acoustic data into images whose contrast is governed by optical absorption.

In the case of optically thin bodies with simple geometries irradiated by short light pulses, solutions to the wave equation for pressure [13], [14], [15], [16] for solid and liquid layers [17], [18], spheres [19], [20], [21], [22], cylinders [17], [23], and ellipsoids [24], [25] yield photoacoustic waveforms with temporal features dependent on the geometry, dimensions, sound speed, and density of the irradiated body. With a knowledge of the density and sound speed of the surrounding fluid, together with a fitting of an experimental waveform to theory, the last three of these acoustic properties of the absorbing body can be determined, thus making the photoacoustic effect an analytical technique. The experimental studies of ultrasonic waves emitted by laser irradiated bodies with symmetry in one, two, and three dimensions have shown excellent agreement with theory. However, there appears to be no investigation, either theoretical or experimental, into the character of the waves produced within the irradiated body although theoretical expressions for determining the waveforms generated internally have been found.

Here, the question of the time dependence of photoacoustic waves found within droplets is addressed. Two problems are presented, the first being determination of the time and space dependence of photoacoustic waves launched from absorption by a spherical region external to the droplet, and second, when the photoacoustic effect is generated by absorption within the droplet itself. Section 2 gives a solution for a fluid sphere surrounded by an optically thin, absorbing layer of a second fluid. The creation of a rapid pressure increase by absorption of a short laser pulse causes compressive waves to enter the droplet resulting in large pressure transients as waves propagate to the center of the droplet. In Section 3 the character of photoacoustic waves generated by absorption of radiation within the sphere itself are investigated. In this case, a series of compressive waves are found along with large rarefactions. In both problems frequency domain expressions are derived, which for the case of equal densities can be Fourier transformed to yield analytical time domain expressions for the sound waves. The Discussion and Conclusions Section notes the possibilities of shock wave generation in spheres even for low laser fluences arising from ultrasonic waves propagating to the origin with ever increasing amplitudes.

## 2 Sphere with a surrounding absorber

In order that the time integral of the pressure should be zero for acoustic waves in two and three dimensions, as noted in Ref. [26], the surrounding layer is given a boundary with a third, non-absorbing fluid that extends from the absorbing spherical region to infinity. It is clear that if the surrounding layer possessed a rigid outer boundary, there would be an overall pressure increase in the sphere and its surroundings long after absorption of the optical radiation so the pressure integral would not vanish. Therefore, a third fluid is taken to surround the absorbing layer. From the point of view of any experiment where the ultrasound within the sphere is of interest, the acoustical character of the third region is irrelevant; the character of sound in this region is thus not considered here. Given the complexity of solving the conservation equation for the photoacoustic effect at two interfaces, the density and sound speed of the infinite layer are taken to be identical to that of the absorbing layer. The part of the problem of interest is thus preserved without the need for carrying out excessive algebraic manipulations.

**Fig. 1:**
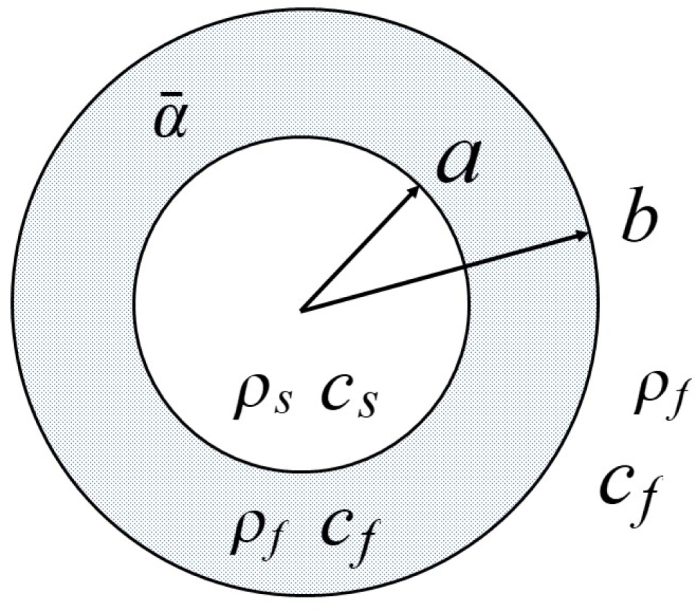
Schematic of an optically transparent fluid sphere of radius a with a density $\rho_{s}$ and sound speed $c_{s}$. The droplet is surrounded by an optically absorbing spherical region of radius b having an optical absorption coefficient $\overset{¯}{\alpha }$, density $\rho_{f}$ and sound speed $c_{f}$. The larger diameter sphere is, in turn, surrounded by a transparent region that extends from b to infinity with the same acoustic properties as the absorbing region.

As shown in Fig. 1, a sphere with density $\rho_{s}$, sound speed $c_{s}$, and radius a is surrounded by an optically thin absorbing layer with an optical absorption coefficient $\overset{¯}{\alpha }$, density $\rho_{f}$, sound speed $c_{f}$, and radius b. The outermost region, which extends from b to infinity is taken to have the same density and sound speed as the absorbing layer. The wave equation for pressure [14] in an inviscid fluid is given by

(1) $$\left(\nabla^{2}-\frac{1}{c^{2}}\frac{\partial^{2}}{\partial t^{2}}\right)p=-\frac{\beta }{C_{P}}\frac{\partial H}{\partial t},$$

where t is the time, c is the sound speed, p is the pressure, β is thermal expansion coefficient, $C_{P}$ is the specific heat capacity, and H is the heating function with dimensions of energy per unit time and volume. Note that the effect of surface tension, which would have the effect of addition of a constant pressure differential [27], is not considered. The sphere is irradiated by an optical beam of intensity $I_{0}$ modulated at a frequency ω. If the heating function and the pressure for the region between a and b is taken to be $H=\overset{¯}{\alpha }I_{0}\exp\left(-i\omega t\right)$ and $p=\overset{\sim }{p}\exp\left(-i\omega t\right)$, respectively, the wave equation becomes a Helmholtz equation of the form

(2) $$\left(\nabla^{2}+k^{2}\right)\overset{\sim }{p}=i\frac{\overset{¯}{\alpha }\beta I_{0}\omega }{C_{P}^{\left(f\right)}},$$

where k=ω∕c. For the absorbing region, the space derivatives in Eq. (2) can be set to zero to give a solution to the inhomogeneous Helmholtz equation for $\overset{\sim }{p}$. When the exponential factor is then included, the frequency domain pressure corresponding to $\overset{\sim }{p}$ becomes

(3) $${\overset{\sim }{P}}^{F}=\frac{i{\overset{\sim }{P}}_{f}}{q\overset{ˆ}{c}}e^{-iq\overset{ˆ}{t}}\;\text{where}\;{\overset{\sim }{P}}_{f}=\frac{\overset{¯}{\alpha }\beta I_{0}c_{f}a}{C_{P}^{\left(f\right)}},$$

where $C_{P}^{\left(f\right)}$ is the specific heat capacity of the fluid in the absorbing region, and a dimensionless frequency q and time $\overset{ˆ}{t}$ have been defined as

(4) $$q=\frac{\omega a}{c}\;\text{and}\;\overset{ˆ}{t}=\frac{c_{s}}{a}t.$$

Thus, for the frequency domain pressures in the sphere ${\overset{¯}{p}}_{s}$, the absorbing layer ${\overset{¯}{p}}_{f}$, and external region ${\overset{¯}{p}}_{e}$, the solutions to the wave equation [28] are

(5) $${\overset{¯}{p}}_{s}={\overset{\sim }{P}}^{F}{\overset{ˆ}{P}}_{s}j_{0}\left(k_{s}r\right)$$

(6) $${\overset{¯}{p}}_{f}={\overset{\sim }{P}}^{F}\left[1+{\overset{ˆ}{P}}_{f}^{\left(1\right)}h_{0}^{\left(1\right)}\left(k_{f}r\right)+{\overset{ˆ}{P}}_{f}^{\left(2\right)}h_{0}^{\left(2\right)}\left(k_{f}r\right)\right]$$

(7) $${\overset{¯}{p}}_{e}={\overset{\sim }{P}}^{F}{\overset{ˆ}{P}}_{e}h_{0}^{\left(1\right)}\left(k_{f}r\right)$$

where the four quantities $\overset{ˆ}{P}$ are constants multiplying the solutions to the homogeneous wave equation, $j_{0}$ is a spherical Bessel function, $h_{0}^{\left(1\right)}$ and $h_{0}^{\left(2\right)}$ are zero order spherical Hankel functions, and where the following dimensionless parameters have been defined for the problem as

(8) $$\overset{ˆ}{c}=\frac{c_{s}}{c_{f}}\;\text{and}\;\overset{ˆ}{\rho }=\frac{\rho_{s}}{\rho_{f}}.$$

Quantities in the sphere have been given subscripts s, those in the absorbing layer f, and those in the external region, e.

The constants multiplying the homogeneous solutions in Eqs. (5), (6), (7) are found by employing the conservation equations for the continuity of pressure and acceleration at interfaces [27], the latter reducing to the ∇p∕ρ. For the interfaces at a and b, these equations are

(9) $${\overset{ˆ}{P}}_{s}j_{0}\left(k_{s}a\right)=1+{\overset{ˆ}{P}}_{f}^{\left(1\right)}h_{0}^{\left(1\right)}\left(k_{f}a\right)+{\overset{ˆ}{P}}_{f}^{\left(2\right)}h_{0}^{\left(2\right)}\left(k_{f}a\right)$$

(10) $${\overset{ˆ}{P}}_{s}j_{0}^{\prime }\left(k_{s}a\right)=\overset{ˆ}{\rho }\overset{ˆ}{c}\left[{\overset{ˆ}{P}}_{f}^{\left(1\right)}h_{0}^{\left(1\right)}{}^{\prime }\left(k_{f}a\right)+{\overset{ˆ}{P}}_{f}^{\left(2\right)}h_{0}^{\left(2\right)\prime }\left(k_{f}a\right)\right]$$

(11) $${\overset{ˆ}{P}}_{e}h_{0}^{\left(1\right)}\left(k_{f}b\right)=1+{\overset{ˆ}{P}}_{f}^{\left(1\right)}h_{0}^{\left(1\right)}\left(k_{f}b\right)+{\overset{ˆ}{P}}_{f}^{\left(2\right)}h_{0}^{\left(2\right)}\left(k_{f}b\right)$$

(12) $${\overset{ˆ}{P}}_{e}h_{0}^{\left(1\right)}{}^{\prime }\left(k_{f}b\right)={\overset{ˆ}{P}}_{f}^{\left(1\right)}h_{0}^{\left(1\right)\prime }\left(k_{f}b\right)+{\overset{ˆ}{P}}_{f}^{\left(2\right)}h_{0}^{\left(2\right)}{}^{\prime }\left(k_{f}b\right).$$

Without the assumption of identical sound speeds and densities for the absorbing and infinite regions extremely complicated expressions for the photoacoustic pressures resulted; consequently, the assumption that these quantities be identical was chosen.

The solution of Eqs. (9), (10), (11), (12) for the four $\overset{ˆ}{P}$ parameters gives lengthy expressions, the only one of interest here being that for ${\overset{ˆ}{P}}_{s}$, which is found^1^ to be

(13) $${\overset{ˆ}{P}}_{s}=\frac{N}{D},$$

$$N=\overset{ˆ}{\rho }\overset{ˆ}{c}\left[h_{0}^{{\left(1\right)}^{\prime }}\left(k_{f}a\right)h_{0}^{\left(1\right)\prime }\left(k_{f}b\right)h_{0}^{\left(2\right)}\left(k_{f}a\right)-h_{0}^{\left(1\right)}{}^{\prime }\left(k_{f}a\right)h_{0}^{\left(1\right)\prime }\left(k_{f}b\right)h_{0}^{\left(2\right)}\left(k_{f}b\right)-\;h_{0}^{\left(1\right)}{}^{\prime }\left(k_{f}a\right)h_{0}^{\left(1\right)}{}^{\prime }\left(k_{f}b\right)h_{0}^{\left(2\right)\prime }\left(k_{f}a\right)+h_{0}^{\left(1\right)}{}^{\prime }\left(k_{f}b\right)h_{0}^{\left(1\right)\prime }\left(k_{f}a\right)h_{0}^{\left(2\right)}{}^{\prime }\left(k_{f}b\right)\right]$$

and

$$D=\left[h_{0}^{\left(1\right)\prime }\left(k_{f}b\right)h_{0}^{\left(2\right)}\left(k_{f}b\right)-h_{0}^{\left(1\right)}\left(k_{f}b\right)h_{0}^{\left(2\right)\prime }\left(k_{f}b\right)\right]\;\times \left[h_{0}^{\left(1\right)}\left(k_{f}a\right)j_{0}^{\prime }\left(k_{s}a\right)-h_{0}^{\left(1\right)}{}^{\prime }\left(k_{f}a\right)j_{0}\left(k_{s}a\right)\overset{ˆ}{\rho }\overset{ˆ}{c}\right].$$

It is convenient to express ${\overset{ˆ}{P}}_{s}$ in terms of the dimensionless parameters

(14) $$k_{f}a=q\overset{ˆ}{c}\;k_{f}b=q\overset{ˆ}{c}\overset{ˆ}{b}\;\overset{ˆ}{b}=b∕a\;\overset{ˆ}{r}=r∕a.$$

After substitution for explicit forms of the spherical Bessel functions, $j_{0}\left(x\right)=\sin\left(x\right)\;\text{/}\;x$, $h_{0}^{\left(1\right)}\left(x\right)=\exp\left(ix\right)\;\text{/}\;\left(ix\right)$, $h_{0}^{\left(2\right)}\left(x\right)=\exp\left(-ix\right)\;\text{/}\;\left(-ix\right)$, and their derivatives into Eq. (13) and simplification of the result, the frequency domain photoacoustic pressure within the sphere is found to be

(15) $${\overset{¯}{p}}_{s}=2{\overset{\sim }{P}}_{f}\left(\frac{\overset{ˆ}{\rho }}{\overset{ˆ}{c}}\right)\frac{\sin\left(q\overset{ˆ}{r}\right)}{q\overset{ˆ}{r}}\left[\frac{\left(1-i\overset{ˆ}{c}q\right)e^{iq}-\left(1-i\overset{ˆ}{c}qb\right)e^{iq\left(\overset{ˆ}{c}b-\overset{ˆ}{c}+1\right)}}{\left[\left(1-\overset{ˆ}{\rho }\right)+iq\left(1+\overset{ˆ}{\rho }\overset{ˆ}{c}\right)\right]\left[1+Re^{2iq}\right]}\right]e^{-iq\overset{ˆ}{t}},$$

where R is an amplitude reflection coefficient for a spherical wave at a spherical interface given by

(16) $$R=\frac{-\left(1-\overset{ˆ}{\rho }\right)+iq\left(1-\overset{ˆ}{\rho }\overset{ˆ}{c}\right)}{\left(1-\overset{ˆ}{\rho }\right)+iq\left(1+\overset{ˆ}{\rho }\overset{ˆ}{c}\right)}.$$

Note that the factor containing R in the denominator of Eq. (15) can be expanded in an infinite series, which in the time domain results in time delays in the pulse sequence within the sphere.

If $\overset{ˆ}{\rho }$ is set to unity, it is possible to obtain the inverse Fourier transform of Eq. (15) in analytic form.^2^ In taking the Fourier transform of the frequency domain pressure it is to be noted that the integration over ω means that ${\overset{\sim }{P}}_{f}$ becomes $\overset{¯}{\alpha }\beta E_{0}c_{f}^{2}\overset{ˆ}{c}∕C_{P}^{\left(f\right)}$ so that the time domain photoacoustic pressure response to a delta function heating pulse becomes

(17) $$p_{s}=-P_{f}\frac{1}{2\overset{ˆ}{r}\left(1+\overset{ˆ}{c}\right)}\overset{\infty }{\underset{n=0}{\sum }}{\left(-R^{\prime }\right)}^{n}\overset{4}{\underset{i=1}{\sum }}F_{i}\left(2n\right),$$

where $P_{f}$, with dimensions of pressure, $R^{\prime }$, the equal density reflection coefficient, and the functions F are given by

(18) $$P_{f}=\frac{\overset{¯}{\alpha }\beta E_{0}c_{f}^{2}}{C_{P}^{\left(f\right)}}\;\;R^{\prime }=\frac{1-\overset{ˆ}{c}}{1+\overset{ˆ}{c}}$$

(19) $$F_{1}\left(n\right)=\left(1+n-\overset{ˆ}{c}-\overset{ˆ}{r}-\overset{ˆ}{t}\right)sgn\left(\overset{ˆ}{r}+\overset{ˆ}{t}-1-n\right)$$

(20) $$F_{2}\left(n\right)=\left(1+n-\overset{ˆ}{c}+\overset{ˆ}{r}-\overset{ˆ}{t}\right)sgn\left(\overset{ˆ}{r}-\overset{ˆ}{t}+1+n\right)$$

(21) $$F_{3}\left(n\right)=\left(-1-n+\overset{ˆ}{c}-\overset{ˆ}{r}+\overset{ˆ}{t}\right)sgn\left(1+\overset{ˆ}{c}\left(\overset{ˆ}{b}-1\right)+n+\overset{ˆ}{r}-\overset{ˆ}{t}\right)$$

(22) $$F_{4}\left(n\right)=\left(-1-n+\overset{ˆ}{c}+\overset{ˆ}{r}+\overset{ˆ}{t}\right)sgn\left(-1-\overset{ˆ}{c}\left(\overset{ˆ}{b}-1\right)-n+\overset{ˆ}{r}+\overset{ˆ}{t}\right)$$

where sgn(x) is the sign function defined as −1, 0, or 1 depending whether x is negative, zero, or positive.

**Fig. 2:**
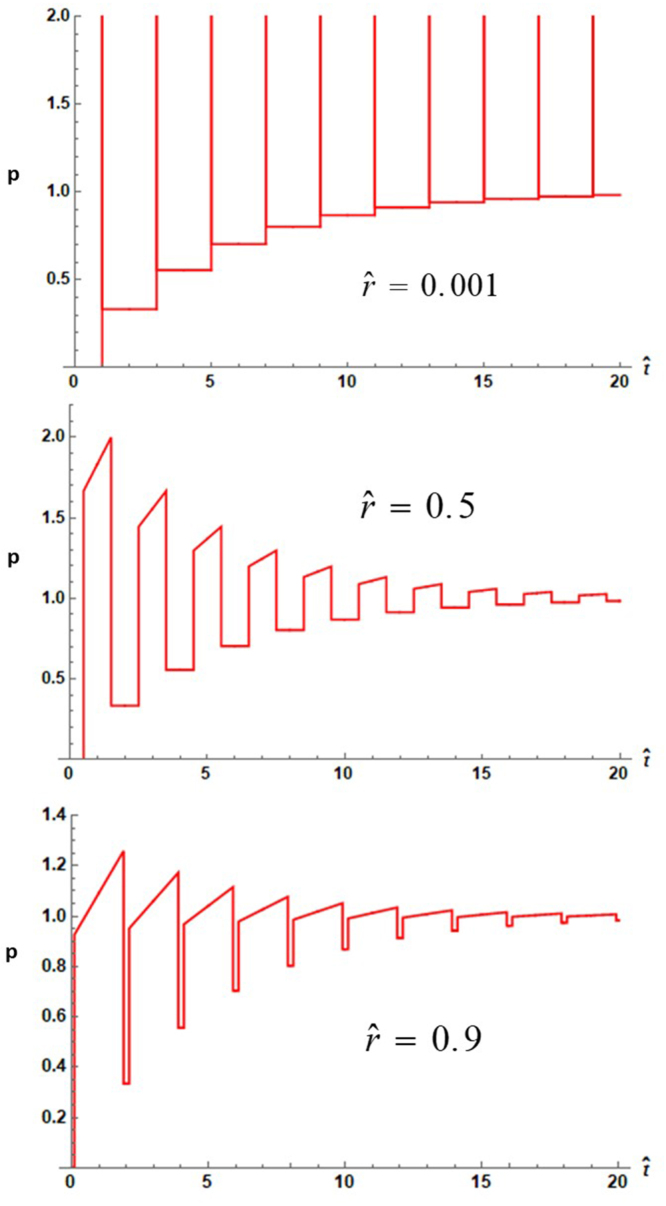
Dimensionless photoacoustic pressure versus dimensionless time from Eq. (17) with 40 terms in the summation for $\overset{ˆ}{c}=5$ with $P_{f}$ set to one. The top plot is truncated since the peak pressure reaches a value of approximately 800.

The results of the calculation for three positions within the droplet are shown in Fig. 2. It can be seen that where $\overset{ˆ}{r}$ is small, the pressure takes on large values, which, given the ${\overset{ˆ}{r}}^{-1}$ dependence of the pressure amplitude in the prefactor of Eq. (17) indicates that the pressure transients are essentially unbounded. Fig. 2 is plotted for a sufficiently large value of $\overset{ˆ}{b}$ that the effects of waves emanating at the interface between the absorbing layer and the external region do not arrive on the time scale plotted. For longer times or smaller values of $\overset{ˆ}{b}$ additional waves appear that have substantial rarefactions. When $\overset{ˆ}{c}$ is given values less than one, e.g. 0.2, for small values of $\overset{ˆ}{r}$ a series of square wave compressions with large spikes of high amplitude compressions and rarefactions appear. When $\overset{ˆ}{r}$ is near unity a series of triangular waves are found. For the case $\overset{ˆ}{c}=1$, $\overset{ˆ}{b}=5$ and $\overset{ˆ}{r}=0.001$, Eq. (17) yields a compressive square wave starting at $\overset{ˆ}{t}=1$ and ending at $\overset{ˆ}{t}=5$, preceded by a compressive spike at $\overset{ˆ}{t}=1$ and followed by a rarefaction spike at $\overset{ˆ}{t}=5$. For larger values of $\overset{ˆ}{b}$, the square wave extends farther out in time to whatever value of $\overset{ˆ}{b}$ that is used.

## 3 Absorbing sphere

**Fig. 3:**
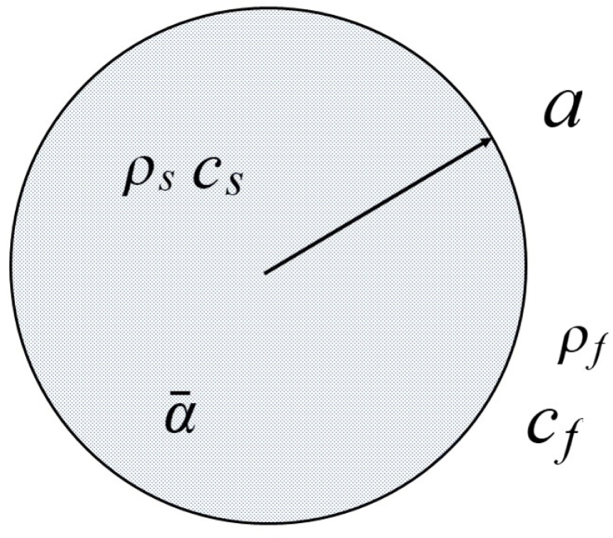
Schematic of an optically thin fluid sphere of radius a with an optical absorption coefficient $\overset{¯}{\alpha }$, density $\rho_{s}$ and sound speed $c_{s}$ surrounded by an infinite medium with density $\rho_{f}$ and sound speed $c_{f}$.

Determination of the photoacoustic effect from an optically thin, absorbing sphere (as shown in Fig. 3) has been reported in Ref. [20] as noted above. The frequency domain photoacoustic pressure inside the sphere is given by

(23) $${\overset{¯}{p}}_{s}={\overset{\sim }{P}}_{s}\frac{i}{q}\left[1+\frac{\overset{ˆ}{\rho }\left(1-i\overset{ˆ}{c}q\right)\left(\sin q\overset{ˆ}{r}∕q\overset{ˆ}{r}\right)}{\left(1-\overset{ˆ}{\rho }\right)\sin q∕q-\cos q+i\overset{ˆ}{\rho }\overset{ˆ}{c}\sin q}\right]e^{-iq\overset{ˆ}{t}},$$

where

(24) $${\overset{\sim }{P}}_{s}=\frac{\overset{¯}{\alpha }\beta I_{0}c_{s}a}{C_{P}^{\left(s\right)}},$$

and where $C_{P}^{\left(s\right)}$ is the specific heat capacity of the fluid in the sphere. Substitution of exponential functions for the sine and cosine functions in Eq. (23) yields

(25) $${\overset{¯}{p}}_{s}={\overset{\sim }{P}}_{s}\left[\frac{i}{q}+\frac{2\overset{ˆ}{\rho }\left(1-i\overset{ˆ}{c}q\right)\left(\sin q\overset{ˆ}{r}∕q\overset{ˆ}{r}\right)e^{iq}}{\left[\left(1-\overset{ˆ}{\rho }\right)+iq\left(1+\overset{ˆ}{\rho }\overset{ˆ}{c}\right)\right]\left[1+Re^{2iq}\right]}\right]e^{-iq\overset{ˆ}{t}},$$

which in the limit where $\overset{ˆ}{\rho }=1$ gives

(26) $${\overset{¯}{p}}_{s}={\overset{\sim }{P}}_{s}\left[\frac{i}{q}+\frac{2\left(1-i\overset{ˆ}{c}q\right)\left(\sin q\overset{ˆ}{r}∕q\overset{ˆ}{r}\right)e^{iq}}{iq\left(1+\overset{ˆ}{c}\right)\left[1+R^{\prime }e^{2iq}\right]}\right]e^{-iq\overset{ˆ}{t}}.$$

As above, it is possible to obtain an analytic form solution for the Fourier transform of Eq. (26). The photoacoustic pressure within the sphere is found to be

(27) $$p_{s}=P_{s}\left\{\frac{sgn\left(\overset{ˆ}{t}\right)}{2}+\frac{1}{2\overset{ˆ}{r}\left(1+\overset{ˆ}{c}\right)}\overset{\infty }{\underset{n=0}{\sum }}{\left(-R^{\prime }\right)}^{n}\left[G_{1}\left(2n+1\right)+G_{2}\left(2n+1\right)\right]\right\},$$

where

(28) $$G_{1}\left(n\right)=\left(\overset{ˆ}{c}-n-\overset{ˆ}{r}+\overset{ˆ}{t}\right)sgn\left(\overset{ˆ}{t}-\overset{ˆ}{r}-n\right)$$

(29) $$G_{2}\left(n\right)=\left(n-\overset{ˆ}{c}-\overset{ˆ}{r}-\overset{ˆ}{t}\right)sgn\left(\overset{ˆ}{t}+\overset{ˆ}{r}-n\right),$$

and

(30) $$P_{s}=\frac{\overset{¯}{\alpha }\beta E_{0}c_{s}^{2}}{C_{P}^{\left(s\right)}}$$

The frequency domain pressure in the surrounding fluid can be found from the acoustic conservation equations at the interface, as above, to be

(31) $${\overset{¯}{p}}_{f}={\overset{\sim }{P}}_{s}\frac{2\left(\sin q-q\cos q\right)∕q}{\overset{ˆ}{r}\left[\left(1-\overset{ˆ}{\rho }\right)+iq\left(1+\overset{ˆ}{\rho }\overset{ˆ}{c}\right)\right]\left[1+Re^{2iq}\right]}e^{-iq\left(\overset{ˆ}{\tau }-1\right)},$$

where $\overset{ˆ}{\tau }=\overset{ˆ}{t}-\overset{ˆ}{c}\left(\overset{ˆ}{r}-1\right)$ is the retarded time from the perimeter of the sphere. Fourier transformation of Eq. (31) into the time domain with $\overset{ˆ}{\rho }=1$ yields

(32) $$p_{f}=P_{s}\frac{1}{2\overset{ˆ}{r}\left(1+\overset{ˆ}{c}\right)}\overset{\infty }{\underset{n=0}{\sum }}{\left(-R^{\prime }\right)}^{n}\left[H_{1}\left(2n+1\right)+H_{2}\left(2n+1\right)\right],$$

where

(33) $$H_{1}\left(n\right)=\left(n+\overset{ˆ}{c}\left(\overset{ˆ}{r}-1\right)-\overset{ˆ}{t}\right)sgn\left(1+n+c\left(\overset{ˆ}{r}-1\right)-\overset{ˆ}{t}\right)$$

(34) $$H_{2}\left(n\right)=\left(n+\overset{ˆ}{c}\left(\overset{ˆ}{r}-1\right)-\overset{ˆ}{t}\right)sgn\left(1-n-c\left(\overset{ˆ}{r}-1\right)+\overset{ˆ}{t}\right).$$

The photoacoustic pressures calculated from Eqs. (27), (32) are found to be identical as $\overset{ˆ}{r}$ approaches one.

**Fig. 4:**
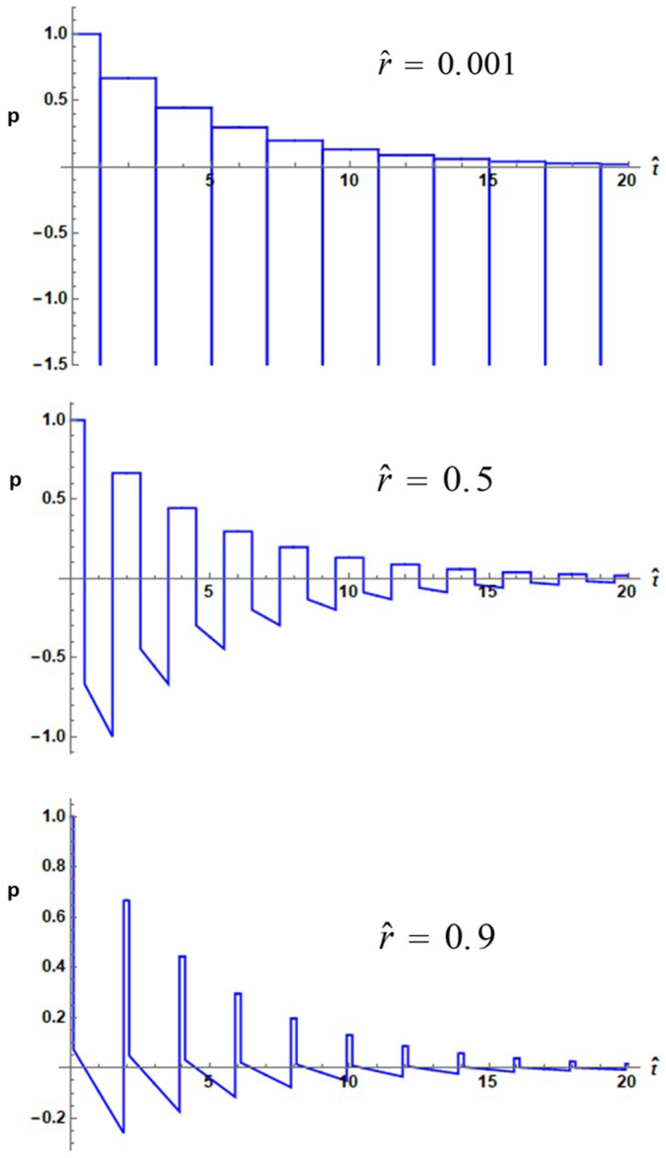
Dimensionless photoacoustic pressure with $P_{s}$ set to unity versus dimensionless time from Eq. (27) with 40 terms in the summation with $\overset{ˆ}{c}=5$. The top plot is truncated as the first peak reaches a value of approximately −800.

Several plots of pressure versus dimensionless time from Eq. (27) are given in Fig. 4. Unlike the case for the externally irradiated sphere, at small values of $\overset{ˆ}{r}$, the transients are high amplitude rarefactions. As $\overset{ˆ}{r}$ approaches unity, the wave takes on the character of a series of repeated N-shaped waves, which appear in time with the sharp spikes in amplitude diminished or non-existent. The series of repeated N-shaped waves with diminishing amplitude resemble the emitted waves found from Eq. (32), as described in Ref. [20].

## 4 Discussion and conclusions

The large amplitude waves reported here follow as a consequence of spherical wave propagation to the center of the droplet, as is explicit in the time domain expressions derived above. Analytic Fourier transforms of the complete frequency domain expressions that include density differences could not be found; however, their numerical Fourier transforms as well as experimental results [19] show that the effect of density differences in the two fluids is to give qualitatively the same waveforms calculated from Eq. (32), but with curvature on the repeated waveforms, as opposed to strictly triangular features, with the curvature becoming more pronounced for each repetition of the initial wave. The origin of the difference in waveform shape for the two cases where the density ratio is unity or departs from this value is that the reflection coefficient R is frequency dependent whereas $R^{\prime }$ is not. Thus when the fluids have identical densities, features of the waveforms repeat in time. For unequal densities the frequency dependence of R results in different degrees of filtering of the different components of the frequency spectrum in the wave at each reflection.

According to Ref. [26], for two and three-dimensional waves the time integral of the pressure must be zero, that is,

(35) ∫p(t)dt=0

**Fig. 5:**
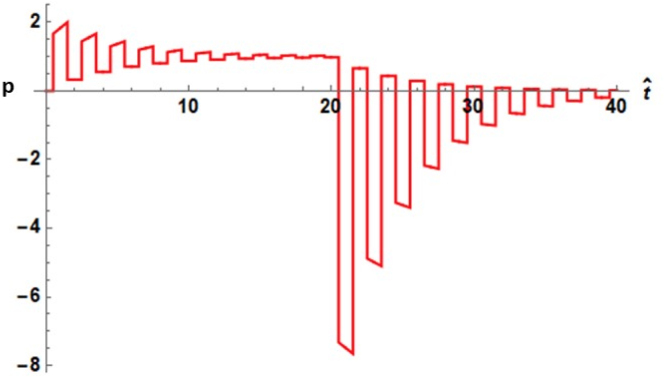
Dimensionless pressure versus $\overset{ˆ}{t}$ from Eq. (17) evaluated with $\overset{ˆ}{c}=5$.

In order to insure that this integral be zero, a non-absorbing layer of fluid that extends from b to infinity was considered. Had the absorbing layer been of finite extent the pressure integral would become a constant. It is easily verified by numerical integration that the integrals of the pressure in the time domain expressions given above vanish, although this is not evident in Fig. 2, which shows only the initial compressive features. However, if large values of $\overset{ˆ}{t}$ are included in the plot so that the wave emanating from the boundary at b becomes visible, a second wave with significant rarefactions is found. Fig. 5 shows an example of this cancellation over the range of $\overset{ˆ}{t}=0$ to 40. The dimensionless pressure numerically integrated over this range gives a value of 0.3; if the integration range is extended to $\overset{ˆ}{t}=80$ the integral becomes $2\times 10^{-6}$.

The pressure amplitudes $P_{f}$ and $P_{s}$ are prefactors in the expressions for the time domain expressions for both problems treated here and multiply the dimensionless amplitudes given in the pressure plots to give the actual photoacoustic pressure. In so far as experimental generation of shock waves within a droplet is concerned, it is easily found that the factors $P_{f}$ and $P_{s}$ for hexane with an absorption of $1\;{\mathrm{mm}}^{-1}$ irradiated by laser with a fluence of $1\;J∕{\mathrm{cm}}^{2}$ results in a pressure of 240atm. This figure presupposes that the laser pulse width be much smaller than the transit time of sound across the droplet. The 240atm figure when multiplied by the peak values of the large transient spikes shown in Fig. 2 suggest that shock wave production is highly likely. A factor to consider however is that the results given here have been derived for a perfect sphere whose index of refraction matches that of the surrounding fluid. Departure from sphericity will change somewhat the time dependence of the spikes and their amplitudes. Any mismatch in index of refraction or departure from the condition that the absorber be optically thin will result in a non-uniform heat deposition within the droplet, either of which will act to reduce the amplitude of the pressure spikes.

In addition to extension of the study of the acoustics of the laser irradiated droplet to the pressure within droplets, part of the motivation for this investigation has been to point out production of large amplitude compressions and rarefactions within a droplet. The possibility exists for manipulating biological objects such as cells placed at the centers of droplets with the high amplitude acoustic waves that have been shown to exist. In any experimental verification of the above results it should be noted that the size of the droplet strongly influences the frequency spectrum of the emitted photoacoustic wave. The experimental investigations referenced in the Introduction Section showed that ultrasonic waves emitted from objects with dimensions of millimeters generated waves with features on the order of microseconds; more recent experiments with micron sized particles [29] showed the ultrasonic waveforms possessed compressions and rarefactions on the nanosecond time scale. The frequency response of the detection is thus governed not only by the duration of the transients as shown in Fig. 2, Fig. 4, but also by the size of the droplet.

## Declaration of Competing Interest

The authors declare that they have no known competing financial interests or personal relationships that could have appeared to influence the work reported in this paper.

## Footnotes

1 Extensive algebraic manipulation is required to solve the four equations; therefore Mathematica was used to find the solution.

2 Extensive algebraic manipulation is required to solve the four equations; therefore Mathematica was used to find the solution.
